# How effective is the comprehensive approach to rehabilitation (CARe) methodology? A cluster randomized controlled trial

**DOI:** 10.1186/s12888-017-1565-y

**Published:** 2017-12-11

**Authors:** Neis Bitter, Diana Roeg, Marcel van Assen, Chijs van Nieuwenhuizen, Jaap van Weeghel

**Affiliations:** 1 Department of Social and Behavioural Sciences, Tranzo Scientific Center for Care and Welfare, Tilburg University, PO Box 90153, 5000 LE Tilburg, The Netherlands; 2 Department of Social and Behavioural Sciences, Methodology and statistics, Tilburg University, PO Box 90153, 5000 LE Tilburg, The Netherlands; 3 GGzE Institute for Mental Health Care, PO BOX 909, 5600 AX Eindhoven, The Netherlands; 4 Department of Sociology, Utrecht University, PO BOX 80140, 3508 TC Utrecht, The Netherlands; 5 Phrenos Centre of Expertise, PO Box 1203, 3500 BE Utrecht, The Netherlands; 6 Parnassia Group, Dijk en Duin Mental Health Centre, PO Box 305, 1900 AH Castricum, The Netherlands

**Keywords:** Rehabilitation, Recovery, Strengths, Severe mental illness, Sheltered facilities

## Abstract

**Background:**

The CARe methodology aims to improve the quality of life of people with severe mental illness by supporting them in realizing their goals, handling their vulnerability and improving the quality of their social environment. This study aims to investigate the effectiveness of the CARe methodology for people with severe mental illness on their quality of life, personal recovery, participation, hope, empowerment, self-efficacy beliefs and unmet needs.

**Methods:**

A cluster Randomized Controlled Trial (RCT) was conducted in 14 teams of three organizations for sheltered and supported housing in the Netherlands. Teams in the intervention group received training in the CARe methodology. Teams in the control group continued working according to care as usual. Questionnaires were filled out at baseline, after 10 months and after 20 months. A total of 263 clients participated in the study.

**Results:**

Quality of life increased in both groups, however, no differences between the intervention and control group were found. Recovery and social functioning did not change over time. Regarding the secondary outcomes, the number of unmet needs decreased in both groups. All intervention teams received the complete training program. The model fidelity at T1 was 53.4% for the intervention group and 33.4% for the control group. At T2 this was 50.6% for the intervention group and 37.2% for the control group.

**Conclusion:**

All clients improved in quality of life. However we did not find significant differences between the clients of the both conditions on any outcome measure. Possible explanations of these results are: the difficulty to implement rehabilitation-supporting practice, the content of the methodology and the difficulty to improve the lives of a group of people with longstanding and severe impairments in a relatively short period. More research is needed on how to improve effects of rehabilitation trainings in practice and on outcome level.

**Trial registration:**

ISRCTN77355880, retrospectively registered (05/07/2013).

## Background

People with serious mental illnesses (SMI) experience numerous problems in their daily lives. Studies on employment, for instance, show that about 10–20% of people with SMI have regular paid employment, 50% work as volunteers or participate in organized day activities and approximately 40% have no paid or unpaid employment at all [1, 2]. Furthermore, a lack of social contacts and loneliness is common among people with SMI [3–5]. Therefore, in addition to medical and psychiatric treatment, these people are in need of services concerning psychiatric rehabilitation and societal participation [1–3].

Over the last two decades, mental health care organizations have applied several psychiatric rehabilitation practices [4, 5]. The goal of these practices is ‘to help individuals with complex, longer term mental health problems to develop the emotional, social and practical skills needed to live, learn and work in the community with the least amount of professional support’ [5–7]. Psychiatric rehabilitation is closely related to the concept of personal recovery. Personal recovery implies a client-oriented definition of recovery in which the emphasis lies more on personal development and growth than on symptom reduction. Important aspects of recovery are: hope, empowerment and the feeling of living a satisfying life despite symptoms of illness [8–16]. While recovery is an individual and subjective process, mental health care organizations can be recovery-oriented. The recovery of clients with SMI can be supported by, among other things, providing psychiatric rehabilitation services [5, 17].

Different approaches to rehabilitation have been developed to help people identify and achieve their own individual goals, including living independently, self-care, gaining and staying in employment, participating in routine educational settings, developing better relationships with their families, and pursuing leisure activities [18–21]. Comprehensive methods exist which focus on the personal goals and wishes of clients. Examples of well-known comprehensive rehabilitation methods are the Boston Psychiatric Rehabilitation (PR) approach [6] and the strengths model [22]. There are also rehabilitation methods which focus on a specific aspect of life, for example, ‘Individual Placement and Support’ (IPS) in which people are supported to gain and stay in competitive employment [23]. Finally, there are methods that aim at improving cognitive functioning or practical skills, e.g., cognitive remediation [24, 25] and cognitive adaptation training (CAT) [26, 27].

Internationally, there is an growing amount of evidence for the effectiveness of the aforementioned interventions on social functioning [5, 7, 19, 20, 23, 28]. Swildens and colleagues [29] found that, among clients who participated in the Boston PR approach, goal attainment and social functioning were significantly higher compared with clients in the control condition. Furthermore, IPS has a strong effect on vocational outcomes [23, 30, 31]. The strengths model is associated with positive results on different outcomes [32–34] including decreased hospitalization and improved quality of life and social functioning [33, 35]. Although research on rehabilitation methods thus shows promising results, their effectiveness remains largely unknown. For example, few randomized controlled trials (RCTs) have been conducted to research the strengths model [32, 36], and several of these studies had methodological limitations such as small sample sizes and inadequate randomization [32, 37]. Furthermore, little is known about the effectiveness of these rehabilitation-oriented practices for clients of sheltered housing facilities [38].

In the Netherlands, a rehabilitation method that is well known and often applied in mental health care is the Comprehensive Approach to Rehabilitation (CARe) methodology. The overall goal of the CARe methodology is to support a client in his/her recovery and to improve his/her quality of life. The central principles of this approach are: realizing goals and wishes; handling vulnerability; and improving the quality of the client’s social environment [39, 40]. The methodology is strongly influenced by the concept of ‘personal recovery’ and by the strengths model [28]. The CARe methodology is used in several mental health care organizations and organizations for sheltered and supported housing. It is suitable for all clients who experience psychosocial problems, regardless of the severity of their impairments or the phase of their recovery process. The CARe methodology is applied by multiple mental health care organizations and organizations for sheltered and supported housing in the Netherlands and abroad. However, no controlled studies have yet been executed on the CARe methodology [41]. The aim of this study was to investigate the effectiveness of the CARe methodology, which was implemented by means of training the professionals of the teams, on personal recovery, quality of life, social functioning, hope, empowerment, self-efficacy beliefs and care needs of people with SMI.

## Methods

### Study design

**Fig. 1:**
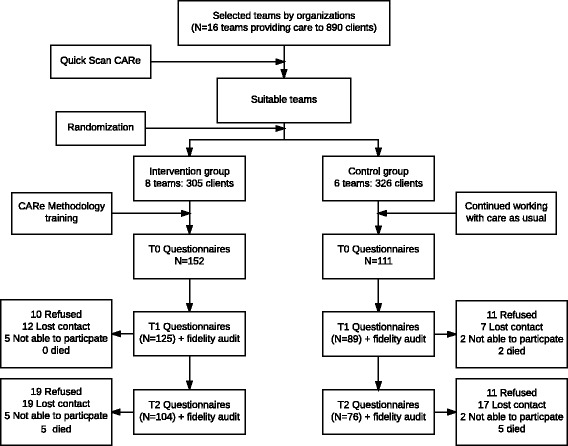
Flowchart of the study. All participants included on T0 were asked to participate again on T1 as well as on T2. Therefore in this flowchart, we report on T1 and T2 the total numbers of dropouts for that moment. ‘Not able to participate’ refers to cases in which a participant was at that moment of measurement not able to understand or fill in the questionnaires due to for example cognitive impairments, psychotic episodes or feelings of anxiety or depression

This study was a two-armed cluster RCT, executed in teams selected from three organizations for sheltered and supported housing in the Netherlands. Randomization was applied at the team level and was stratified by organization. Professionals in the intervention group received the CARe training program; teams in the control group continued to offer ‘care as usual’. The professionals and researchers were aware of the allocation of the conditions; clients could not be blinded but it was not explicitly pointed out to them which condition they were in. Outcomes were measured at baseline (T0), and at 10 (T1) and 20 months (T2) afterwards (see Fig. 1) [41].

The study received ethical approval from the Medical Research Ethics Committee of the Elisabeth Hospital in Tilburg (NL41169.008.12). The trial registration number is ISRCTN77355880 (http://www.controlled-trials.com/ISRCTN77355880).

### Setting

In dialogue with the national supported housing alliance, we selected three sheltered housing organizations with an articulated interest in training their employees in the CARe methodology that were invited to participate. These organizations, which were all situated in (semi-)urban areas, provide ‘sheltered housing’, including permanent supervision in (semi-) individual or group facilities, and supported independent living services including home-based support. Teams often provide both type of services and consist of social workers and nurses. The organizations are not responsible for the psychiatric treatment of their clients, which is provided by external mental health care organizations.

### Intervention

#### The CARe methodology

The central aim of the CARe methodology is improving the quality of life of people with a psychological or social vulnerability. The CARe methodology addresses this aim in three ways: (1) realizing the client’s wishes and goals; (2) handling vulnerability and reinforcing strengths; and (3) obtaining access to desired environments and improvement of the quality of the client’s living environment and social networks. The CARe methodology is strongly influenced by the following concepts: the presence approach [42], the personal recovery movement [11], and the strengths model of case management [22, 43–45].

The CARe methodology consists of the following six steps:

1. Building a relationship with the client
  In the CARe methodology the relationship between client and worker is seen as the basis of offering professional support. Central elements of this relation are: safety, active support and personal meeting. The presence approach of Baard (46) focusing on an equal relationship and frequent attendance is used.
2. Drawing up a ‘strengths assessment’
  The aim of using the strengths assessment is to create insight in the experiences, strengths and resources of a client on four personal (i.e., self-care, health, meaningfulness and social relations) and four life domains (i.e., living, working, learning and recreating). The experiences, strengths and resources of the past and in the present time are drawn up together with the client.
3. Helping the client to formulate his/her wishes and goals
  The wishes of a client are the starting point. The worker supports the client in exploring and formulating his wishes. Based on the strengths assessment of step 2, formulates wishes and translates these in one or two concrete goals with support of the worker.
4. Helping the client to make a ‘recovery worksheet’
  In a ‘recovery worksheet’ concrete steps and activities are described to achieve the goals from step 3. It includes the role of others in the support system of the client.
5. Helping the client to execute the recovery worksheet
  During the execution of the plan, handling and accepting vulnerabilities are topics a worker gives attention to. Besides that, there is attention for strengths of the clients. The professional’s support in seeking connections in the environment, for example by improving the accessibility of a desired environment and creating support in the society.
6. Adjusting the recovery worksheet
  The recovery worksheet is a ‘living document’. The trajectory and the goals are evaluated and changed when needed. It is a repeating cyclic process that helps the client to grow in putting rehabilitation goals into action and adjust plans when required.

#### Training and coaching

The training consisted of seven meetings, i.e., three full-day theory meetings and four half-day ‘training on-the-job’. Qualified trainers from a specialized training institute conducted these meetings. Table 1 shows the topics which were addressed in the training. After finishing the training, teams receive coaching every 4 to 6 weeks. During these coaching sessions the professionals discuss an example of a client in a methodical way. A trained CARe coach guided these sessions.

**Table 1:** Content of the CARe methodology training

• Theoretical principles of the CARe methodology: recovery, presence, strengths oriented working, social participation and using environmental resources.
• Building a partnership with a client and the basic principles of supporting clients.
• Connecting to the recovery process of a client.
• Inventorying the client’s wishes and strengths and seeing possibilities to realise these.
• Formulating concrete goals with the client.
• Draw up plans: a personal plan for the client and a support plan for the professional.
• Introduction to the CARe Toolkit with specific tools for specific cases, for example an instrument to map a client’s social network.

#### Care as usual

The teams in the control group did not receive the CARe training program. The workers in those teams continued working according to ‘care as usual’. This implied working according to common practice. Several differences exist between care as usual and the CARe methodology. The most important difference between teams in the intervention group and teams in the control group teams was that the control teams did not work with the ‘strengths assessment’ and the ‘recovery worksheet’, which are seen as the central instruments of the CARe methodology. Besides that they were not be supported by the ‘CARe coaching meetings’. Finally, teams in the control group were asked not to implement new practices oriented on recovery, rehabilitation or strengths during the study.

### Recruitment of teams

Because rehabilitation practices are common in sheltered and supported housing facilities in the Netherlands, it was impossible to include teams that did not work according to any rehabilitation method at all. However, to study the effects of the CARe methodology in a randomized design, teams using as least as possible rehabilitation methodology were needed. These teams were selected in two steps. First, each participating organization was asked to make a selection of possible teams suitable for this study, teams in which (most of) the workers did not receive training in a rehabilitation method before or in which the use of rehabilitation principles were downgraded due to, for example, turnover of staff or poor implementation. Teams that were trained completely in the CARe methodology were excluded from this study. Second, a researcher (NB) interviewed the team leaders and made a definitive selection by means of the ‘Quick Scan CARe’, an instrument developed to map the general use of the CARe methodology principles in a team. Only teams with a very low score on this quick scan were included in the study and randomly allocated to the intervention or the control group. In total, 14 teams providing care to 631 clients were selected to participate in the study (Fig. 1).

### Recruitment of participants

Recruitment of clients took place between September 2012 and June 2013. The researchers sent an information brochure to all clients 18 years or older who were receiving services from one of the included teams. Subsequently, clients were approached by the researcher (NB) or via the staff for participation in the study. Participants were asked to give their informed consent in writing before the start of the first interview. Each participant was informed about his or her right to withdraw from the study at any time. Clients with too little knowledge of the Dutch language to fill in the questionnaire and/or clients who were unable to give informed consent or to participate in the study due to cognitive impairment or clinical symptoms were excluded.

### Model fidelity

After 10 and 20 months of the training program, a comprehensive ‘CARe methodology fidelity audit’ was performed for all the teams to check the implementation level and the contrast between intervention and control teams. The audits were performed by the first author (NB) and a CARe expert; both received training in this audit from the developer. In this audit, the model fidelity was scored by means of interviews with three clients, three workers, team leader and CARe-coach and by a random check of three client files.

### Outcomes

The following self-reported questionnaires were used to measure the outcomes.

#### Primary outcomes

Quality of life, social functioning and personal recovery were the primary outcomes, relating to the main goals of the CARe methodology. Quality of life was measured using the Manchester Short Appraisal (MANSA). The MANSA (α = 0.74) consists of 12 subjective items with a seven-point Likert scale (‘could not be worse’–‘could not be better’) [39, 40]. Social functioning was measured using the Social Functioning Scale (SFS). The scale (α = 0.80) consists of 19 items and four checklists on seven domains: social engagement/withdrawal, interpersonal behavior, pro-social activities, recreation, independence-competence, independence-performance and employment/occupation [46]. Personal recovery was measured by the Mental Health Recovery Measure (MHRM). The MRHM is a self-report instrument with 30 items. The MHRM is a reliable and valid instrument. The instrument comprises three subscales: ‘self-empowerment’ (α = 0.90), ‘learning and new potentials’(α = 0.86) and ‘spirituality’ (α = 0.94). All items are rated using a five-point Likert scale that ranges from ‘strongly disagree’ to ‘strongly agree’ [13]. For quality of life and recovery, we calculated the mean score of the full scale; for social functioning, we calculated a sum score.

#### Secondary outcomes

Empowerment, hope, self-efficacy beliefs and need for care were the secondary outcomes. Empowerment was measured by the Dutch Empowerment Scale. This scale consists of 40 items distributed over six domains: professional help (α = 0.81), social support (α = 0.87), own wisdom (α = 0.89), belonging (α = 0.74), self-management (α = 0.74) and involvement in community (α = 0.81). The items are scored on a five-point Likert scale ranging from ‘strongly disagree’ to ‘strongly agree’ [47, 48]. Hope was measured by the Herth Hope Index (HHI), consisting of 12 four-point Likert scale items ranging from ‘strongly disagree’ to ‘strongly agree’. The Dutch version of the HHI consists of two factors, each of six items: ‘view on life and future’ (α = 0.8) and ‘self-confidence and inner strength’ (α = 0.69) (overall α = 0.84) [49, 50]. Health-related self-efficacy beliefs were measured by the Mental Health Confidence Scale (MHCS). This scale has 16 items with a six-point Likert scale (‘totally no confidence’–‘full confidence’). The instrument has three subscales: optimism (six items, α = 0.87), coping (seven items, α = 0.76) and advocacy (three items, α = 0.93) [51, 52]. Need for care was measured by the 27-item version of the Camberwell Assessment of Needs Short Appraisal Schedule (CANSAS). With this instrument the client can score a health or social need as ‘no need’, ‘fulfilled need’ or ‘unfulfilled need’ [53]. Concerning empowerment, hope and self-efficacy beliefs, we calculated the overall mean score. Regarding needs for care, we calculated the total amount of ‘unmet needs’.

#### Additional and control outcomes

The following demographic variables were measured: age, gender, marital status, employment status and living situation. Additionally, the key workers of the participating clients were asked to answer questions regarding the psychiatric diagnosis (DSM IV) of the client and the amount of contact they had with the client (hours per day and/or week). Psychiatric symptomatology was measured by use of the client-rated Brief Symptom Inventory (BSI). This is a 53-item self-report questionnaire (α = 0.96). This instrument assesses clinical symptoms during the past week. The items are rated using a five-point scale, ranging from ‘not at all’ to ‘extremely’. The BSI has nine subscales: somatisation, obsessive-compulsive, interpersonal sensitivity, depression, anxiety, hostility, phobia, paranoia and psychoticism [54]. The client-rated Recovery Promoting Relationship Scale (RPRS) (α = 0.80) was used to measure to what extent the client experiences the relationship with his or her key worker as supporting his/her recovery. The scale consists of 24 items with a four-point Likert scale ranging from 1 (strongly disagree) to 4 (strongly agree) and with five indicating not applicable [55]. The workers’ knowledge of recovery was measured by use of the staff-rated Recovery Knowledge Inventory (RKI) (α = 0.80). The RKI consists of 20 items (scored on a five-point Likert scale ranging from strongly disagree to strongly degree) [55, 56].

### Statistical analysis

Sample size was calculated taking into account the design effect (due to group randomization) and the expected effect size. The sample size calculation was based on the measures with the strongest expected effect size according to comparable studies [48, 51]. Within the duration of the study of 20 months these were: empowerment (d = 0.38) and hope (d = 0.50). The design effect used is estimated to be 1.5 based on an average cluster size of 38 clients and an intra-cluster correlation (ICC) of 0.013. Based on the effect size of empowerment (d = 0.38; the lowest of the two above mentioned), this design effect, and a planned power of 0.80 using a two-sided test, a sample of 128 clients per condition was calculated to be needed. Assuming a loss of 20% due to follow up, we aimed to recruit 160 = clients per condition.

Descriptive statistics for both intervention and control groups were computed separately and differences were tested using *t*-tests for continuous and *χ* ^*2*^-tests for discrete variables. Subsequently, we used a linear mixed modeling (procedure MIXED in SPSS 22) to test our main hypotheses. The advantage of mixed models over more traditional approaches ((M)ANOVA) is that linear mixed modeling can accommodate missing values and time-varying covariates.

For each of the outcome variables, several mixed models were tested. All models had the same covariance structure, a random effect of ‘team’ taking into account team effects and a compound symmetry covariance structure for time. We first estimated the ICC for both team and participants within one team. Subsequently, two models were fitted, one with only main effects of time and intervention, and the second with the time × intervention interaction as well. The interaction tests the effectiveness of the intervention over time. The interaction was tested two-tailed by comparing the −2 log likelihood of the models. The two models were fitted both without covariates and with covariates (age, gender, having a partner, symptoms, amount of support, recovery-promoting relationship and recovery knowledge of the professionals). Because effects of covariates were observed for all variables, only the results of the model with covariates were reported. If the interaction was statistically significant at .05, we checked using simple slope analysis how the effect of the intervention group differed from the control group over time.

## Results

### Teams

The overall mean score on the model fidelity (% possible achievable points) at T1 was 53.4% for the intervention group and 33.4% for the control group. At T2 this was 50.6% for the intervention group and 37.2% for the control group.

### Clients

In total, 263 clients agreed to participate in the study: 152 in the intervention group and 111 in the control group. At the first follow up, 81% were still included; at the second follow up this was 68%. The intervention group consisted of a significantly higher number of clients who lived in a sheltered living facility (*p* < .001) using a significantly higher amount of support (p < .001) in comparison with the control group. No other differences were observed (see Table 2).

**Table 2:** Client characteristics at baseline (*N* = 263)

	Intervention (*N* = 152)		Control (*N* = 111)	
Characteristic	Number	Percent	Number	Percent
Mean Age (SD)	50.76 (14.29)		49.36 (13.25)	
Male	98	65	72	65
Having a partner	18	12	19	17
Nationality				
Born in the Netherlands	129	85	95	86
Other	23	15	16	14
Type of care^a^				
Sheltered living	125	83	65	59
Supported independent living	26	17	46	41
Work situation				
Paid work	4	3	5	5
Sheltered work	11	7	12	11
No work	82	54	66	60
Voluntary work	40	26	24	22
Retired	11	7	3	3
Amount of support^a^				
>daily	97	69	47	47
>weekly	25	18	30	30
once a week	10	7	21	21
<weekly	8	6	2	2
Primary outcomes	M (SD)		M (SD)	
Quality of life (*N* = 262)	4.08 (.70)		3.93 (.67)	
Social functioning (*N* = 263)	112.13 (24.76)		109.57 (23.21)	
Personal recovery (*N* = 262)	3.52 (.55)		3.41 (.48)	
Secondary outcomes	M (SD)		M (SD)	
Hope (*N* = 262)	2.91 (0.38)		2.84 (.38)	
Empowerment (*N* = 242)	3.64 (0.48)		3.60 (.49)	
Self-efficacy (*N* = 240)	4.41 (0.91)		4.36 (.76)	
Needs (*N* = 254)				
Unmet needs	3.95 (3.16)		4.45 (2.83)	
Met needs	8.34 (3.16)		7.80 (3.22)	
No needs	14.53 (3.33)		14.59 (3.42)	
Covariates	M (SD)		M (SD)	
BSI (*N* = 257)	.71 (.62)		0.82 (.63)	
RPRS (*N* = 230)	3.49 (.61)		3.62 (.53)	

^a^type of facility and amount of support differed significantly (*p* < .001) between the groups. On other variables, the groups did not differ significantly

### Preliminary analysis

Means, standard deviations, sample sizes and Cohen’s d for all measures at T0, T1 and T2 are shown in Table 3. On T1, a small to medium significantly different change score between the intervention and control group was found on both quality of life (Cohen’s d = .373; *p* = .01) and unmet needs (Cohen’s d = .316; *p* = .03) in favor of the intervention group. On T2, no differences were found.

**Table 3:** Means (SD) at baseline and at 10 and 20 months assessments

	T0		T1			T2		
Primary outcomes	Intervention	Control	Intervention	Control	Cohen’s d^a^	Intervention	Control	Cohen’s d
Quality of life	4.08 (.70) *N* = 152	3.93 (.67) *N* = 110	4.15 (.66) *N* = 124	3.89 (.70) *N* = 88	.0.373 t(210) = 2.71 *p* = .01	4.57 (.95) *N* = 104	4.53 (.75) *N* = 76	0.051 t(178) = .33 *p* = .74
Social functioning	112.13 (24.76) *N* = 152	109.57 (23.21) *N* = 111	107.86 (26.92) *N* = 125	108.57 (23.89) *N* = 89	−.028 t(212) = −.20 *p* = .84	111.78 (22.93) *N* = 104	115.87 (24.96) *N* = 76	−.170 t(178) = −1.14 *p* = .26
Personal recovery	3.52 (.55) *N* = 152	3.41 (.48) *N* = 110	3.55 (.44) *N* = 125	3.44 (.57) *N* = 89	.212 t(212) = 1.59 *p* = .11	3.58 (.46) *N* = 104	3.46 (.51) *N* = 76	.259 t(178) = 1.74 *p* = .08
Secondary outcomes								
Hope	2.91 (.38) *N* = 152	2.84 (.38) *N* = 110	2.89 (.34) *N* = 123	2.84 (.39) *N* = 89	.148 t(210) = 1.09 *p* = .28	2.92 (.35) *N* = 103	2.87 (.36) *N* = 75	.143 t(176) = .95 *p* = .35
Empowerment	3.64 (.48) *N* = 139	3.60 (.49) *N* = 103	3.67 (.39) *N* = 111	3.57 (.54) *N* = 82	.215 t(141) = 1.42 *p* = .16	3.67 (.41) *N* = 99	3.67 (.49) *N* = 73	.070 t(170) = .44 *p* = .66
Self-efficacy	4.41 (.91) *N* = 139	4.36 (.76) *N* = 101	4.51 (0.62) *N* = 112	4.35 (.81) *N* = 81	.227 t(144) = 1.59 *p* = .14	4.43 (.73) *N* = 97	4.42 (.73) *N* = 70	.004 t(165) = .03 *p* = .98
Unmet needs	3.95 (3.16) *N* = 149	4.45 (2.83) *N* = 106	3.16 (2.3) *N* = 117	4.0 (3.04) *N* = 89	.316 t(159) = −2.14 (*P* = .03)	2.18 (2.31) *N* = 101	2.85 (.79) *N* = 72	.252 t(171) = −1.72 *p* = .09

^a^Cohen’s d corresponds to the difference in change scores from baseline between the intervention and control group. Cohen’s d is positive if it is in the expected direction

### Mixed modeling

The ICC for ‘team’ was .284 for social functioning and varied between .000 and .030 for the other variables. Therefore, a random effect of team was only included in the analysis of social functioning. The participants ICCs were between .571 and .675, demonstrating much larger systematic individual differences in the outcomes (Tables 4 and 5, row ‘ICC team’ and ‘ICC participants’).

**Table 4:** Mixed modeling analysis testing the effect of the CARe methodology on primary outcomes

	Quality of life			Personal recovery				Social functioning		
ICC team^a^	.030			.007				.284		
ICC participants	.602			.652				.673		
Model	Test	P	95%CI	Test	P	95%CI		Test	P	95%CI
Time	F = 22.37	.00		F = 2.87	.06			F = 2.05	.13	
T1	B = .04	.45	−.07–.15	B = .03	.44	−.04–.10		B = .38	.80	−2.53-3.30
T2	B = .51	.00	.35–.66	B = .12	.02	.02–.22		B = 4.22	.05.	.01–8.42
Intervention	B = .06	.52	−.14–.27	B = .09	.10	−.02–.20		B = 4.67	.07	−.32–9.66
Intervention x time^b^	Χ^2^ = 4.46	.11		Χ^2^ = 1.28	.53			Χ^2^ = 4.64	.10	
Covariates^c^										
Age	B = .00	.18	.00–.01	B = .00	.92	−.00–.00	B = −.96	.00	−1.13--.78	
Gender	B = .08	.31	−.08–.25	B = −.08	.17	−.20–.03	B = 5.23	.05	−.07–10.53	
Partner	B = .06	.51	−.11–.22	B = .09	.12	−.02–.21	B = 5.4	.03	.50–10.37	
Symptoms	B = −.55	.00	−.66--.44	B = −.31	.00	−.39--.24	B = −9.72	.00	−13.06—6.39	
Amount of support	B = −.01	.79	−.09–.07	B = −.01	.64	−.07–.04	B = 4.70	.00	2.29–7.11	
Recovery knowledge team	B = −.20	.29	−.58–.17	B = −.10	.44	−.34–.15	B = −11.28	.03	−21.64--.93	
Recovery promoting relationship	B = .33	.00	.21–.44	B = .24	.00	.16–.32	B = 4.71	.01	1.22–8.19	

^a^Intra-Class Correlation for team and participants

^b^Effect of the intervention. The chi-square values are values of the deviance or likelihood ratio test

^c^The effects of the included covariates

**Table 5:** Mixed modeling analysis testing the effect of the CARe methodology on secondary outcomes

	Hope			Empowerment			Self-efficacy			Unmet Needs		
ICC team^a^	.03			.00			.01			.03		
ICC participants	.59			.57			.67			.48		
	Test	P	95% CI	Test	P	95% CI	Test	P	95% CI	Test	P	95% CI
Time	F = 1.80	.16		F = 1.51	.23		F = .32	.73		F = 10.07	.00	
T1	B = .00	.96	−.06–.06		.14	−.12–.02		.45	−.07–.15		.24	−.82–.21
T2	B = −08	07	.00–.16	B = −.05	.70	−.08–.11	B = .04	.99	−.16–.16	B = −.31	.00	−2.34- -.91
Intervention	B = .03	.49	−.07–.13	B = .02	.96	−.09–.09	B = .00	.98	−.17–.18	B = −1.63	.65	−.69–.43
Intervention x time^b^	Χ^2^ = .22	.90		Χ^2^ = 1.99	.37		Χ^2^ = 3.63	.16		Χ^2^ = .73	.70	
Covariates^c^												
Age	B = −.00	.80	.00–.00	B = .00	.07	−.00–.01	B = .00	.85	−.01–.01	B = −.01	.19	−.03–.01
Gender	B = −.13	.00	−.22--.04	B = −.13	.01	−.23--.03	B = −.30	.00	−.48--.11	B = −.48	.11	−1.08–.12
Partner	B = .09	.05	.001–.18	B = .02	.72	−.08–.12	B = .16	.09	−.03–.34	B = −.12	.74	−.83–.59
Symptoms	B = −.23	.00	−.29--.17	B = −.31	.00	−.38---.24	B = −.70	.00	−.82--.58	B = 2.48	.00	2.04–2.90
Amount of support	B = −.03	.21	−.07–.02	B = −.01	.65	−.06–.04	B = .00	.93	−.08–.09	B = −.09	.56	−.41–.23
Recovery knowledge team	B = −.02	.84	−.22–.18	B = .01	.95	−.22–.23	B = −.29	.14	−.68–.09	B = −1.09	.18	−2.66-.49
Recovery promoting relationship	B = .15	.00	.09–.21	B = .38	.00	.32–.45	B = .29	.00	.16–.42	B = −.49	.03	−.94--.04

^a^Intra-Class Correlation for team and participants

^b^Effect of the intervention. The chi-square values are values of the deviance or likelihood ratio test

^c^The effects of the included covariates

The effect of the intervention team at T1 and T2 was not different from that of care as usual-team (Tables 4 and 5 row ‘intervention x time’). Quality of life (Table 4) increased (B = .51 (*p* < .001)) and the amount of unmet needs (Table 5) decreased significantly (B = .31 (p < .001)) in both groups. The CARe training program intervention had no effect on the outcomes (Table row ‘intervention’). The results retained after controlling for background variables (age, gender, having a partner, symptoms, amount of support, recovery-promoting relation and recovery knowledge of professionals) (Tables 4 and 5). Concerning the influence of background variables, BSI and RPRS had a respectively negative and positive effect on all outcomes. Age had a negative effect on social functioning. Gender (male) had a positive effect on hope, empowerment and self-efficacy. Having a partner had an effect on social functioning and hope. The amount of support and recovery knowledge of the team had a respectively positive and negative effect on social functioning.

## Discussion

We examined the effectiveness of training teams of professionals in the CARe methodology on clients of sheltered and supported housing services. Clients improved on quality of life and amount of unmet needs. However, clients of the intervention group did not improve more than clients of teams in the control group measured after 10 and 20 months. This indicates that, in this study, the CARe methodology did not lead to better rehabilitation for clients of supported housing facilities.

There are three relevant discussion points. First, although the CARe training program was provided as meant, and a difference in model fidelity was measured between the control and intervention teams 10 and 20 months after the training, the overall fidelity of the CARe methodology in the intervention teams was limited: it did not exceed 60% at both times. Although, we cannot be sure that higher implementation is possible as there are no other studies on the CARe methodology, there is a change that we cannot ignore that a higher fidelity in CARe would lead to better outcomes. Implementation is a consistent problem in (mental) health care research [57–59]. Barriers in an implementation process can occur at organizational, team and individual levels [60, 61]. In our study, all participating organizations went through reorganizations and budget cuts during the research period, which may have negatively influenced the implementation process on all levels. Participating staff members mentioned factors such as changes in staff and management, a negative work climate and lack of practical and moral support from the organization. Nevertheless, in future research more attention is needed on how this methodology can be implemented more effectively and on methods that can be used to properly monitor and control this implementation process.

A second explanation for our findings might be the characteristics of the CARe methodology itself. Earlier research on other rehabilitation approaches indicated that elements of effective psychiatric rehabilitation are: focusing on the specific skills that are needed in a certain environment and actual access to that desired environment as soon as possible [62]; integrating rehabilitation and psychiatric treatment; and combining skills training and offering support [62, 63]. In the CARe methodology, these aspects are not elaborated explicitly. Nevertheless, much is still unknown on how people with SMI can be supported in their rehabilitation successfully. In order to develop psychiatric rehabilitation and the CARe methodology, it is necessary to conduct more research on the specific efficacious elements of rehabilitation practices [64, 65].

Third, the participating clients might have such severe impairments that this intervention is not strong enough to support them in their recovery and participation. Some studies on psychiatric rehabilitation interventions showed small positive results; these all concerned methods focusing on a selective group of motivated clients with concrete goals [29, 30]. In the CARe methodology, motivation and being capable to formulate goals were not eligibility requirements. Besides that, the intervention group consisted of relatively more clients of a sheltered facility. This may indicate that the group consisted of more vulnerable clients than the control group. However, in none of the outcomes and control variables on baseline significant differences were found between both groups. Thus, although we cannot exclude that group differences in type of accommodation affected the results, our data do not indicate that this is the case. Despite this, it is encouraging that the quality of life of clients participating in this study increased in the total group, although none of the other outcomes improved over time (personal recovery, societal participation, hope, empowerment and self-efficacy). This might indicate that it takes more time and effort to increase recovery and participation for these people. More research is needed on how to support this specific group of people with long-term impairments of whom several have lost their motivation and goals in life [57].

This study is the first effect study on the CARe methodology and one of the few studies with a control group on a comprehensive rehabilitation method or strengths based approach [25, 38]. This study is of high relevance because recovery and rehabilitation oriented care has become increasing important for mental health care organizations, especially nowadays as de-institutionalization and participation in society is increasingly being encouraged [14, 43, 67]. Strength of the study is that a large and diverse group of clients with long-term SMI participated, a group that is often difficult to reach in research. The fidelity assessment is another strength giving a clear indication of the implementation rate that was achieved by training the teams in the intervention group.

Although it is a strength that this study was executed in real care settings, this has also led to some limitations. First, as rehabilitation and recovery oriented working is increasingly common practice in mental health care, it was not possible to select teams with no experience in this respect. Even though we controlled the selection process by using a quick scan, we cannot guarantee that the control condition was totally blank. Another weakness is the fact that the interviewers and fidelity auditors were not blinded. Furthermore, the targeted recruitment was not achieved and the attrition rate was somewhat higher than expected. Finally, because the achieved sample size was lower than the planned sample size, the actual power of our analyses was lower than intended (0.64 instead of 0.8).

## Conclusions

This is the first study on the effectiveness of the CARe methodology. And one of the few studies with a control group on a comprehensive rehabilitation method or strengths based approach executed in a sheltered facility for people with long-term severe impairments. An extensive training program in the CARe methodology for teams of sheltered and supported housing facilities did not lead to more improvement in clients on quality of life, personal recovery and social functioning, served by these teams compared with clients of teams that did not receive such training. Nevertheless, clients in both groups improved on quality of life and amount of unmet needs. The difficulty of implementation of rehabilitation methods and the complexity of changing lives of persons with longstanding and severe problems are important explaining factors. It is recommended to conduct more research on how to overcome these difficulties in order to enlarge the quality of life of people with long-term and severe mental illness.

## Abbreviations

BSI: Brief Symptom Inventory
CANSAS: Camberwell Assessment of Needs Short Appraisal Schedule
CARe: Comprehensive Approach to Rehabilitation
HHI: Herth Hope Index
ICC: Intra Class Correlation
MANSA: Manchester Short Appraisal (MANSA)
MHCS: Mental Health Confidence Scale
MHRM: Mental Health Recovery Measures
RCT: Randomized Controlled Trial
RPRS: Recovery Promoting Relationship Scale
SFS: Social Functioning Scale
SMI: Severe Mental Illness
